# New insights into DNA methylation signatures: *SMARCA2* variants in Nicolaides-Baraitser syndrome

**DOI:** 10.1186/s12920-019-0555-y

**Published:** 2019-07-09

**Authors:** Eric Chater-Diehl, Resham Ejaz, Cheryl Cytrynbaum, Michelle T. Siu, Andrei Turinsky, Sanaa Choufani, Sarah J. Goodman, Omar Abdul-Rahman, Melanie Bedford, Naghmeh Dorrani, Kendra Engleman, Josue Flores-Daboub, David Genevieve, Roberto Mendoza-Londono, Wendy Meschino, Laurence Perrin, Nicole Safina, Sharron Townshend, Stephen W. Scherer, Evdokia Anagnostou, Amelie Piton, Matthew Deardorff, Michael Brudno, David Chitayat, Rosanna Weksberg

**Affiliations:** 1 Genetics and Genome Biology, The Hospital for Sick Children, Toronto, Ontario M5G 1X8 Canada; 2 Division of Genetics, Department of Pediatrics, McMaster University, Hamilton, Ontario L8S 4L8 Canada; 3 Department of Genetic Medicine, Munroe-Meyer Institute, University of Nebraska Medical Center, Omaha, NE USA; 4 Genetics Program, North York General Hospital, Toronto, Ontario M2K 1E1 Canada; 5 Department of Paediatrics, University of Toronto, Toronto, Ontario M5S 3H7 Canada; 6 Department of Pediatrics, UCLA, Los Angeles, CA 90095 USA; 7 Division of Clinical Genetics, Children’s Mercy Hospital, Kansas City, MO 66111 USA; 8 Division of Pediatric Clinical Genetics, University of Utah School of Medicine, Salt Lake City, UT 84132 USA; 9 Service de génétique clinique, Département de génétique médicale, maladies rares, médecine personnalisée, Unité INSERM U1183, Université Montpellier, CHU Montpellier, 34000 Montpellier, France; 10 Division of Clinical and Metabolic Genetics, The Hospital for Sick Children, Toronto, Ontario M5G 1X8 Canada; 11 Department of Pediatrics, University of Toronto, Toronto, Ontario M5S 1A1 Canada; 12 AP-HP, Department of Genetics, Hôpital Robert Debré, 75019 Paris, France; 13 University of Missouri Kansas City, School of Medicine, Kansas City, MO 64108 USA; 14 Division of Clinical Genetics, Children’s Mercy Hospital, Kansas City, MO 64108 USA; 15 Department of Pediatrics, Children’s Mercy Hospital, Kansas City, MO 64108 USA; 16 Department of Health, Government of Western Australia, Genetic Services of Western Australia, Perth, WA Australia; 17 Department of Molecular Genetics, University of Toronto, Toronto, Ontario M5S 1A1 Canada; 18 The Centre for Applied Genomics, The Hospital for Sick Children, Toronto, Ontario M5G 1X8 Canada; 19 McLaughlin Centre, University of Toronto, Toronto, Ontario M5S 1A1 Canada; 20 Holland Bloorview Kids Rehabilitation Hospital Toronto, Toronto, Ontario M4G 1R8 Canada; 21 Institute of Medical Science, University of Toronto, Toronto, Ontario M5S 1A1 Canada; 22 Institut de Génétique et de Biologie Moléculaire et Cellulaire, 67400 Illkirch, France; 23 Laboratoire de Diagnostic Génétique, Nouvel Hôpital Civil, Hôpitaux Universitaires de Strasbourg, 67000 Strasbourg, France; 24 Division of Genetics, The Children’s Hospital of Philadelphia, Philadelphia, PA 19104 USA; 25 The Department of Pediatrics, The Perelman School of Medicine, The University of Pennsylvania, Philadelphia, PA 19104 USA; 26 Centre for Computational Medicine, The Hospital for Sick Children, Toronto, Ontario M5G 1X8 Canada; 27 Department of Computer Science, University of Toronto, Toronto, Ontario M5S 1A1 Canada; 28 Prenatal Diagnosis and Medical Genetics Program, Mount Sinai Hospital, Toronto, Ontario M5G 1X5 Canada; 29 Institute of Medical Sciences, University of Toronto, Toronto, Ontario M5S 1A8 Canada

**Keywords:** SMARCA2, NCBRS, DNA methylation, Signature, Epigenomics, Chromatin remodeling, BAF complex, SWI/SNF, VUS

## Abstract

**Background:**

Nicolaides-Baraitser syndrome (NCBRS) is a neurodevelopmental disorder caused by pathogenic sequence variants in *SMARCA2* which encodes the catalytic component of the chromatin remodeling BAF complex. Pathogenic variants in genes that encode epigenetic regulators have been associated with genome-wide changes in DNA methylation (DNAm) in affected individuals termed *DNAm signatures*.

**Methods:**

Genome-wide DNAm was assessed in whole-blood samples from the individuals with pathogenic *SMARCA2* variants and NCBRS diagnosis (*n* = 8) compared to neurotypical controls (*n* = 23) using the Illumina MethylationEPIC array. Differential methylated CpGs between groups (DNAm signature) were identified and used to generate a model enabling classification variants of uncertain significance (VUS; *n* = 9) in *SMARCA2* as “pathogenic” or “benign”. A validation cohort of NCBRS cases (n = 8) and controls (*n* = 96) demonstrated 100% model sensitivity and specificity.

**Results:**

We identified a DNAm signature of 429 differentially methylated CpG sites in individuals with NCBRS. The genes to which these CpG sites map are involved in cell differentiation, calcium signaling, and neuronal function consistent with NCBRS pathophysiology. DNAm model classifications of VUS were concordant with the clinical phenotype; those within the *SMARCA2* ATPase/helicase domain classified as “pathogenic”. A patient with a mild neurodevelopmental NCBRS phenotype and a VUS distal to the ATPase/helicase domain did not score as pathogenic, clustering away from cases and controls. She demonstrated an intermediate DNAm profile consisting of one subset of signature CpGs with methylation levels characteristic of controls and another characteristic of NCBRS cases; each mapped to genes with ontologies consistent with the patient’s unique clinical presentation.

**Conclusions:**

Here we find that a DNAm signature of *SMARCA2* pathogenic variants in NCBRS maps to CpGs relevant to disorder pathophysiology, classifies VUS, and is sensitive to the position of the variant in *SMARCA2*. The patient with an intermediate model score demonstrating a unique genotype-epigenotype-phenotype correlation underscores the potential utility of this signature as a functionally relevant VUS classification system scalable beyond binary “benign” versus “pathogenic” scoring. This is a novel feature of DNAm signatures that could enable phenotypic predictions from genotype data. Our findings also demonstrate that DNAm signatures can be domain-specific, highlighting the precision with which they can reflect genotypic variation.

**Electronic supplementary material:**

The online version of this article (10.1186/s12920-019-0555-y) contains supplementary material, which is available to authorized users.

## Background

Hundreds of genes encoding epigenetic regulators, known as epigenes, are critical for normal development [1]. Specific types of epigenes, such as chromatin remodelers and histone modifying enzymes, initiate and maintain numerous developmental processes by targeting cell-type-specific regulatory genes [2]. Pathogenic sequence variants in many epigenes cause a variety of genetic disorders characterized by intellectual disability (ID) and disruption of normal growth [1, 3–5]. Our group has demonstrated that many of the disorders caused by pathogenic variants in epigenes are associated with functionally relevant DNAm signatures i.e. characteristic gene-specific changes in DNAm in blood cells. These signatures have been particularly informative for elucidating the pathophysiology of each disorder and for classifying sequence variants as pathogenic or benign. A variety of epigenes have now been identified to demonstrate such signatures including Nuclear Receptor Binding SET Domain Protein 1 (*NSD1*) in Sotos syndrome [6], Lysine Methyltransferase 2D (*KMT2D*) in Kabuki syndrome [7, 8], Chromodomain-helicase-DNA-binding protein 7 (*CHD7*) in CHARGE syndrome [8], Lysine-Specific Demethylase 5C (*KDM5C*) in non-syndromic intellectual disability [9], Chromodomain Helicase DNA Binding Protein 8 (*CHD8*) in autism spectrum disorders (ASD) [10], and DNA Methyltransferase 1 (*DNMT1*) in adult-onset autosomal dominant cerebellar ataxia with deafness and narcolepsy (ADCA-DN) [11]. Gene-specific DNAm signatures are likely to exist for many disorders caused by dysregulation of the epigenetic machinery.

Elucidation of DNAm signatures has significant potential for clinical translation. Whole-exome and targeted diagnostic sequencing can identify sequence variants in genes known to be associated with specific disorders. While some variants are clearly identified as pathogenic or benign, a significant proportion are reported as variants of uncertain significance (VUS). Establishing the pathogenicity of these variants can be challenging. In rare disorders this is particularly difficult as many variants have not previously been reported. In silico tools can be used to predict the effects of novel genomic variants on protein function, but they have many limitations. The accuracy of these predictions is impacted by the location of the variant in certain protein domains [12], the strength of evolutionary conservation of the genomic position [13], and overfitting by using the same variant in both training and evaluation of these tools [13]. In this context, DNAm signatures provide a novel functional classification method with significant potential to improve the output of the genome diagnostics.

Nicolaides-Baraitser syndrome (NCBRS; [MIM# 601358]), is a rare (prevalence < 1/1,000,000) epigene disorder characterized by coarse facial features, sparse hair, seizures, microcephaly, small stature, prominent interphalangeal joints, and ID. NCBRS was first reported as a distinct clinical entity in 1993 by Paola Nicolaides and Michael Baraitser, and soon after was identified in other patients [14–16]. NBCRS is caused by pathogenic missense variants in *SMARCA2* (SWI/SNF Related, Matrix Associated, Actin Dependent Regulator of Chromatin, Subfamily A, Member 2; GenBank NG_032162.2) [17], the core catalytic subunit of the mammalian BAF (BRG1- or HBRM-associated factors) chromatin remodeling complex, also known as the SWI/SNF complex. The BAF complex is a key regulator of neurological development and is also involved in maintenance of neuronal function [18–20]. SMARCA2 hydrolyzes ATP, facilitating nucleosome remodeling at target sites, allowing recruitment of other transcriptional regulators [21, 22]. An exome sequencing study of 10 patients with NCBRS established the etiological role of *SMARCA2* missense variants in the disorder [17]. The authors noted that all pathogenic sequence variants were de novo heterozygous missense variants in the ATPase/helicase domain, indicating that these changes may not impair BAF complex assembly but rather disrupt ATPase function possibly acting in a dominant negative manner [17]. To date, the vast majority of *SMARCA2* pathogenic variants in individuals with NCBRS have mapped to the ATPase/C-terminal helicase domain [17]; only two cases with typical NCBRS phenotypes have been reported to harbor missense variants distal to this domain [23, 24]. Sequence variants in other BAF complex genes are associated with other neurodevelopmental disorders including *SMARCC1*/*2*, *PBRM1*, *ARID1A*/*B* and *SMARCA4* in ASD, *PBRM1* and *ARID1B* in schizophrenia, *SMARCB1* in Kleefstra syndrome, and *ARID1A/B, SMARCA4, SMARCB1*, and *SMARCE1* Coffin-Siris syndrome (CSS) [19, 21]. CSS has substantial clinical overlap with NCBRS [22, 25–27]; due to this overlap some individuals with NCBRS have been misclassified as CSS [28]. More functional and cost-effective diagnostics would greatly aid in differential diagnosis of these cases, ending the diagnostic odyssey for these families.

Here, we generated a DNAm signature associated with NCBRS by comparing patient samples with pathogenic variants in the *SMARCA2* gene to neurotypical controls. We then used the signature to generate a model facilitating classification of VUS in *SMARCA2* as “pathogenic” or “benign”. Gene ontology analysis of the genes overlapping the signature CpG sites identified functions and pathways relevant to NCBRS pathophysiology. Most importantly, we found one case of NCBRS with classic syndromic features but mild neurodevelopmental findings demonstrating a partial DNAm signature. This partial signature was composed of two subsets of CpG sites, one with methylation values characteristic of controls and the other typical of NCBRS profiles. Further the genes these CpG subsets mapped to were consistent with the patient’s specific clinical phenotype.

## Methods

### Research participants

Informed consent was obtained from all research participants according to the protocol approved by the Research Ethics Board of the Hospital for Sick Children (REB# 1000038847). Cases were recruited through the Division of Clinical and Metabolic Genetics at the Hospital for Sick Children, Toronto, Ontario; Children’s Hospital of Philadelphia, Pennsylvania, USA; North York General Hospital, Toronto, Ontario; Primary Children’s Hospital, Salt Lake City, Utah; Children’s Mercy Hospital, Kansas City, Kansas; Hôpitaux Universitaires de Strasbourg, Strasbourg, France; and Prevention Genetics, USA.

Our study cases consisted of individuals with *SMARCA2* variants (*n* = 17) of whom 12 had a clinical diagnosis of NCBRS. Clinically, the NCBRS cases encompassed the variable spectrum of disorder severity (detailed clinical data found in Additional file 2: Table S1). SMARCA2_5 and SMARCA2_11 have been previously published as NBS24 and NBS26 [17], SMARCA2_1 has also been previously described by our group [29]. Unique features noted in the cohort included ophthalmologic abnormalities, such as unilateral retinal detachment in SMARCA2_1, bilateral infantile glaucoma in SMARCA2_4 and myopia in SMARCA2_12 and SMARCA2_14. SMARCA2_12 was different from other reported cases in the mild degree of ID; she is a 16-year-old with normal growth parameters, seizures, learning disability and attention deficit-hyperactivity disorder, who is enrolled to begin college with good social functioning. Clinical photos of SMARCA2_12 are not available as per parental wishes, but she displayed facial coarsening with full lips, a wide mouth and lower lip eversion. Hair was not sparse but rather slow-growing, curly and coarse in quality.

#### NCBRS-SMARCA2 DNAm signature cases

**Fig. 1:**
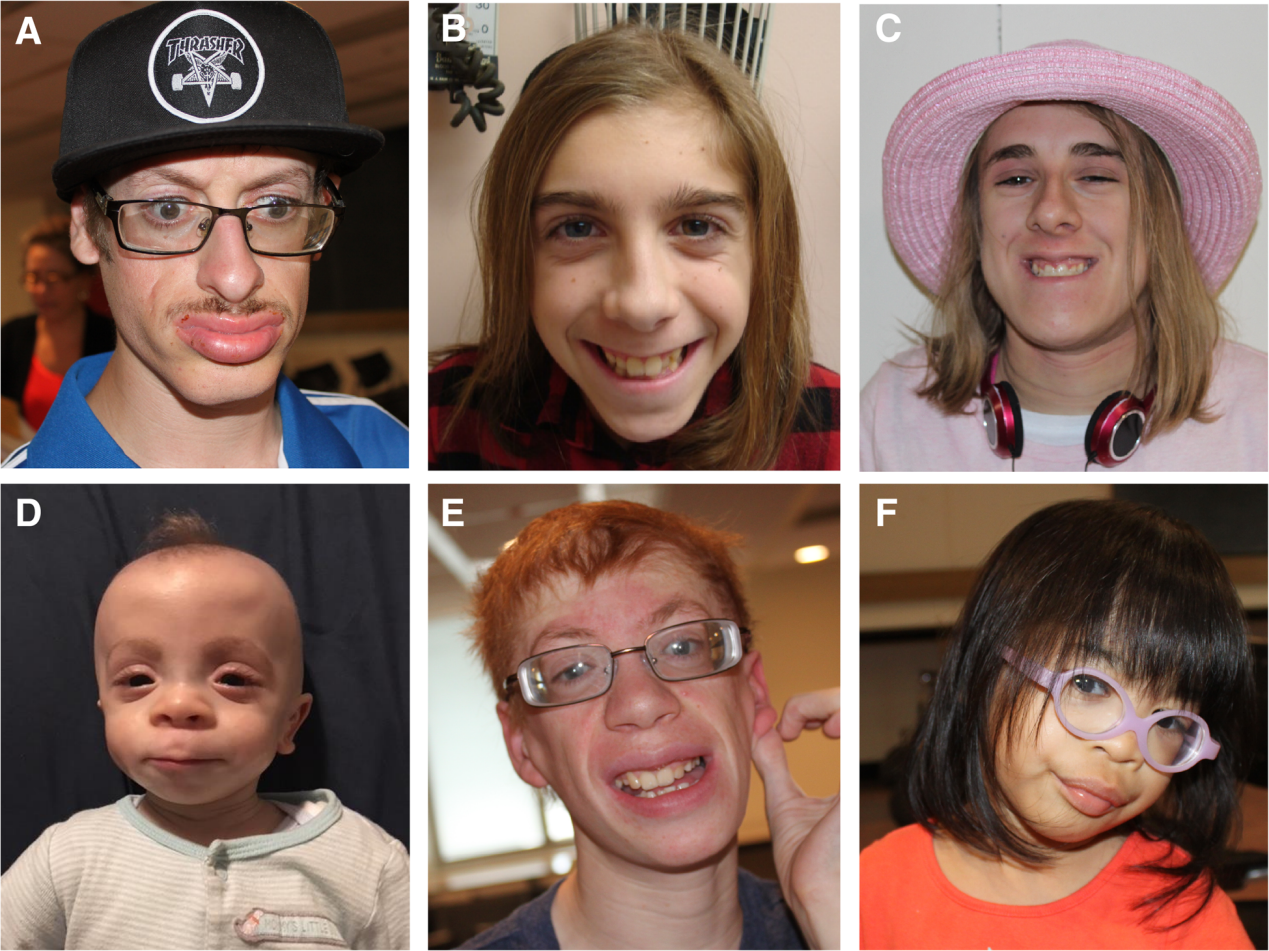
Clinical photographs of selected study patients with a clinical diagnosis of Nicolaides-Baraitser syndrome**.** SMARCA2_1 (**a**), SMARCA2_2 (**b**) and SMARCA2_6 (**c**) were part of the signature derivation case group and share the coarse facial features, thick eyebrows, progressive eversion of the lower lip and prognathism associated with NCBRS. These features are most pronounced in the eldest individual, SMARCA2_1 (**a**), and are known to progress with age. SMARCA2_10 (**d**), SMARCA2_14 (**e**), and SMARCA2_4 (**f**) also show phenotypic features consistent with NCBRS, have variants of uncertain significance in SMARCA2 and were part of the SMARCA2 test variant group

Individuals with pathogenic missense variants in the *SMARCA2* ATPase/helicase domain (as determined by ACMG guidelines by the referring clinical laboratory) and a clinical diagnosis of NCBRS (*n* = 8) were used to generate the DNAm signature (Table 1). The eight individuals in the signature derivation cohort had classic features of NCBRS, with progressive facial coarsening with age (Fig. 1). Sparse hair and malar hypoplasia were particularly evident in infancy (Fig. 1d) with eversion of the lower lip and prognathism emerging more in adulthood (Fig. 1a). Apart from the craniofacial characteristics, poor growth and feeding, seizures, absent or delayed speech, variable ID, and behavioral disturbances (ex. decreased inhibitions, self-aggression, compulsive behavior, and sensory sensitivities in some patents) continue to be common features of the syndrome in the cohort. ASD and ADHD were formally diagnosed in two individuals (Additional file 2: Table S1).

**Table 1:** Variant information and selected clinical data for samples with *SMARCA2* sequence variants

Sample ID	Variant	Inheritance	PolyPhen Prediction Effect (score)	SIFT prediction effect	Mutation Taster prediction effect	CADD score	ExAC Total frequency	Diagnosis	ACMG classification	NCBRS-*SMARCA2* score
**SMARCA2_1**	c.3493C > A, p.Gln1165Lys	de novo	Probably damaging (0.924)	Deleterious	Disease causing	22.9	–	NCBRS	Pathogenic	0.37
**SMARCA2_2**	c.3209 T > A, p.Leu1070Gln	–	Probably damaging (0.998)	Deleterious	Disease causing	29.3	–	NCBRS	Likely pathogenic	0.27
SMARCA2_4	c.2639 C > T, p.Thr880Ile	–	Probably damaging (0.999)	Deleterious	Disease causing	28.2	–	NCBRS	VUS	0.25
**SMARCA2_5**	c.2648C > T, p.Pro883Leu	–	Probably damaging (0.999)	Deleterious	Disease causing	28.9	–	NCBRS	Likely pathogenic	0.24
**SMARCA2_6**	c.2486C > T, p.Thr829Ile	de novo	Probably damaging (0.999)	Deleterious	Disease causing	29.1	–	NCBRS	Pathogenic	0.22
**SMARCA2_7**	c.2264A > G, p.Lys755Arg	–	Probably damaging (0.997)	Deleterious	Disease causing	33.0	–	NCBRS	Pathogenic	0.36
**SMARCA2_8**	c.3623C > G, p.Ser1208Cys	de novo	Probably damaging (0.999)	Deleterious	Disease causing	27.0	–	NCBRS	Likely pathogenic	0.32
**SMARCA2_9**	c.2348C > G, p.Ser783Trp	de novo	Probably damaging (1.0)	Deleterious	Disease causing	34.0	–	NCBRS	Likely pathogenic	0.22
SMARCA2_10	c.2564G > C, p.Arg855Pro	de novo	Probably damaging (0.999)	Deleterious	Disease causing	28.7	–	NCBRS	VUS	0.24
**SMARCA2_11**	c.2255G > C, p.Gly752Ala	–	Probably damaging (0.999)	Deleterious	Disease causing	27.9	–	NCBRS	Likely pathogenic	0.27
SMARCA2_12	c.3849G > T, p.Trp1283Cys	de novo	Probably damaging (0.999)	Tolerated	Disease causing	34.0	–	NCBRS	VUS	−0.04
SMARCA2_14	c.2558G > T, p.Gly853Val	–	Probably damaging (0.83)	Deleterious	Disease causing	29.7	–	NCBRS	VUS	0.29
SMARCA2_15	c.400G > A, p.Val134Ile	–	Probably damaging (0.84)	Tolerated	Disease causing	22.3	0.00001648	–	VUS	−0.25
SMARCA2_16	c.674A > C, p.Gln225Pro	–	Benign (0)	Tolerated	Benign	14.7	0.0001821	–	Benign	−0.32
SMARCA2_17	c.689A > C, p.Gln230Pro	–	Benign (0)	Tolerated	Polymorphism	12.7	0.0003702	–	Benign	−0.31
SMARCA2_18	c.695A > C, p.Gln232Pro	–	Benign (0)	Tolerated	Polymorphism	12.7	0.0006005	–	Benign	−0.30
SMARCA2_19	c.1878-3 T > C, p.Gly626,	–	–	–	–	-	-	–	Benign	−0.24

Cases used to generate the NCBRS-*SMARCA2* DNAm signature are in bold. CADD score > 20 indicates a variant in the top 1% of deleterious variants in the human genome, > 30 in the top 0.1%. ACMG classification was made by the referring clinical laboratory for each sample. NCBRS-*SMARCA2* score was generated in this study based on the DNAm signature (see Methods). Detailed clinical data are presented in Additional file 2: Table S1

#### SMARCA2 variant classification cases

Individuals with *SMARCA2* variants (VUS [*n* = 5]; benign [*n* = 4]) were classified using the DNAm signature. Four individuals had a clinical diagnosis of NCBRS with VUS in *SMARCA2*. The remaining five cases (VUS [*n* = 1]; benign [n = 4]) had no obvious phenotypic features of NCBRS and were identified through exome sequencing. Available phenotypic details can be found in Additional file 2: Table S1.

#### Signature controls

The DNAm signature was derived using age- and sex-matched neurotypical controls (*n* = 23) (Additional file 2: Table S2) obtained from the POND Network, The Hospital for Sick Children, and The University of Michigan (Dr. Greg Hanna) [30]. Neurotypical was defined as healthy and developmentally normal by using formal cognitive/behavioral assessments (samples from POND and The University of Michigan) or via physician/parental screening questionnaires (Hospital for Sick Children).

### DNAm microarray data processing

Whole-blood DNA samples were bisulfite converted using the EpiTect Bisulfite Kit (EpiTect PLUSBisulfite Kit, QIAGEN). The sodium bisulfite converted DNA was then hybridized to the Illumina Infinium Human MethylationEPIC BeadChip to interrogate over 850,000 CpG sites in the human genome at The Center for Applied Genomics (TCAG), SickKids Research Institute, Toronto, Ontario, Canada. Sample groups were divided equally among chips, then randomly assigned a chip position. All signature-derivation cases and controls were run in the same batch of chips and reagents. The *minfi* Bioconductor package in *R* was used to preprocess data including quality control, Illumina normalization and background subtraction followed by extraction of β values. Standard quality control metrics in *minfi* were used, including median intensity QCplots, density plots, and control probe plots: all showed good data quality for all samples. Probes were removed with detection flaws (*n* = 801), probes near SNPs with minor allele frequencies above 1% (*n* = 29 958), cross-reactive probes [31] (*n* = 41 975), probes with raw beta = 0 or 1 in > 0.25% of samples (*n* = 15), non-CpG probes (n = 2 925), X and Y chromosome probes (*n* = 57 969) for a total of *n* = 774 521 probes remaining for differential methylation analysis. The accession number for the DNAm data for the cases, controls, and test variants reported in this paper is GEO: GSE125367.

### NCBRS*-SMARCA2* DNAm signature

We defined a DNAm signature of differentially methylated sites in whole-blood DNA of NCBRS cases with pathogenic *SMARCA2* variants (*n* = 8) compared with age- and sex-matched control samples (*n* = 23). We termed this an NCBRS-*SMARCA2*-specific DNAm signature since it was derived specifically on *SMARCA2* pathogenic variant samples within the ATPase/helicase domain with an NCBRS clinical diagnosis. We used the cell-type proportion estimation tool in *minfi* based on Illumina EPIC array data from FACS sorted blood cells [32]. This indicated a significant decrease in CD4+ T cells and a significant increase in monocytes in the signature cases (Additional file 2: Table S3). Since these cell types are highly correlated, we used only estimated monocyte proportion in the regression model as it was the more significantly different. As there is a substantial effect of age on DNAm [33], we used only cases and controls older than 2 years of age to generate the NCBRS signature. We verified that the signature could classify controls under 2 years using four one-year-old neurotypical controls. CpG sites with significantly different methylation values between signature cases and controls were identified using *limma* regression, with age, sex, and estimated monocyte proportion as covariates. We identified a DNAm signature with a Benjamini-Hochberg adjusted *p*-value< 0.05 and a |Δβ| > 0.10 (10% methylation difference) comprised of 429 probes (Additional file 2: Table S4).

### NCBRS-*SMARCA2* score

We developed a classification model using the NCBRS-*SMARCA2* DNAm signature. At each of the 429 signature CpGs, a median DNAm level was computed across the NCBRS cases (n = 8) used to generate the signature, resulting in a reference profile. Similarly, a robust median-DNAm reference profile for the signature controls (n = 23) was created. The classification of each test variant or control DNAm sample was based on extracting a vector *Bsig* of its DNAm values in the signature CpGs, and comparing *Bsig* to the two reference profiles computed above. NCBRS-*SMARCA2* score was defined as: *NCBRS*-*SMARCA2 score = r(B*_*sig*_*, NCBRS profile) – r(B*_*sig*_*, control profile)* [1] where *r* is the Pearson correlation coefficient. A classification model was developed based on scoring each new DNAm sample using the NCBRS-*SMARCA2* Score: a test sample with a positive score is more similar to the NCBRS reference profile based on the signature CpGs, and is therefore classified as “pathogenic”; whereas a sample with a negative score is more similar to the control-blood reference profile, and is classified as “benign”. The classification is implemented in *R*. To test specificity, EPIC array data from 94 additional neurotypical controls were scored and classified. To test sensitivity, publically available EPIC array data from NCBRS cases with different variants [34] (GSE116992) were scored and classified. All were classified correctly, demonstrating 100% sensitivity and specificity of the signature. Publicly available sorted blood cell type data [32] (GSE110554) were also scored and classified.

### Pathway analysis

The list of 429 DNAm signature CpG sites was submitted to GREAT (Genomic Regions Enrichment of Annotations Tool) for gene ontology (GO) enrichment analysis [35]. Enrichment of the gene list in each GO term is calculated using a foreground/background hypergeometric test over genomic regions; we used the set of CpG sites after *minfi* probe quality control (*n* = 774 521) as a background set. Terms with two or more gene hits were reported (Additional file 2: Tables S5-S8).

### Differentially methylated regions in the signature-derivation cohort

The bumphunting [36, 37] design matrix accounted for the potential confounding effects of sex, age, and blood cell-type factors (estimated monocyte proportion). The analysis considered CpGs with |Δβ| > 10% between cases and controls as candidates for the DMRs, with gaps < 500 bp between neighboring CpGs. Statistical significance was established using 1,000 randomized bootstrap iterations, as is recommended. The resulting DMRs were post-filtered to retain only those with *p*-value< 0.01 and a length (number of consecutive CpGs) of a least four.

### DNAm validation by sodium bisulfite pyrosequencing

An independent analysis of DNAm was performed for NCBRS-*SMARCA2* signature cases (*n* = 8) and a subset of matched controls (n = 8) using sodium bisulfite pyrosequencing. Controls 2, 4, 10, 13, 14, 17, 18, and 24 were used, as they mostly closely matched the age and sex of the NCBRS cases. These assays were designed using QIAGEN Assay Design Software v1.0.6 to target-specific CpGs identified by the microarray experiment (Additional file 1: Table S5). Pyrosequencing was done using the PyroMark Q24 system and Pyrosequencing Gold Reagents (QIAGEN).

## Results

### NCBRS-*SMARCA2* DNA methylation signature

**Fig. 2:**
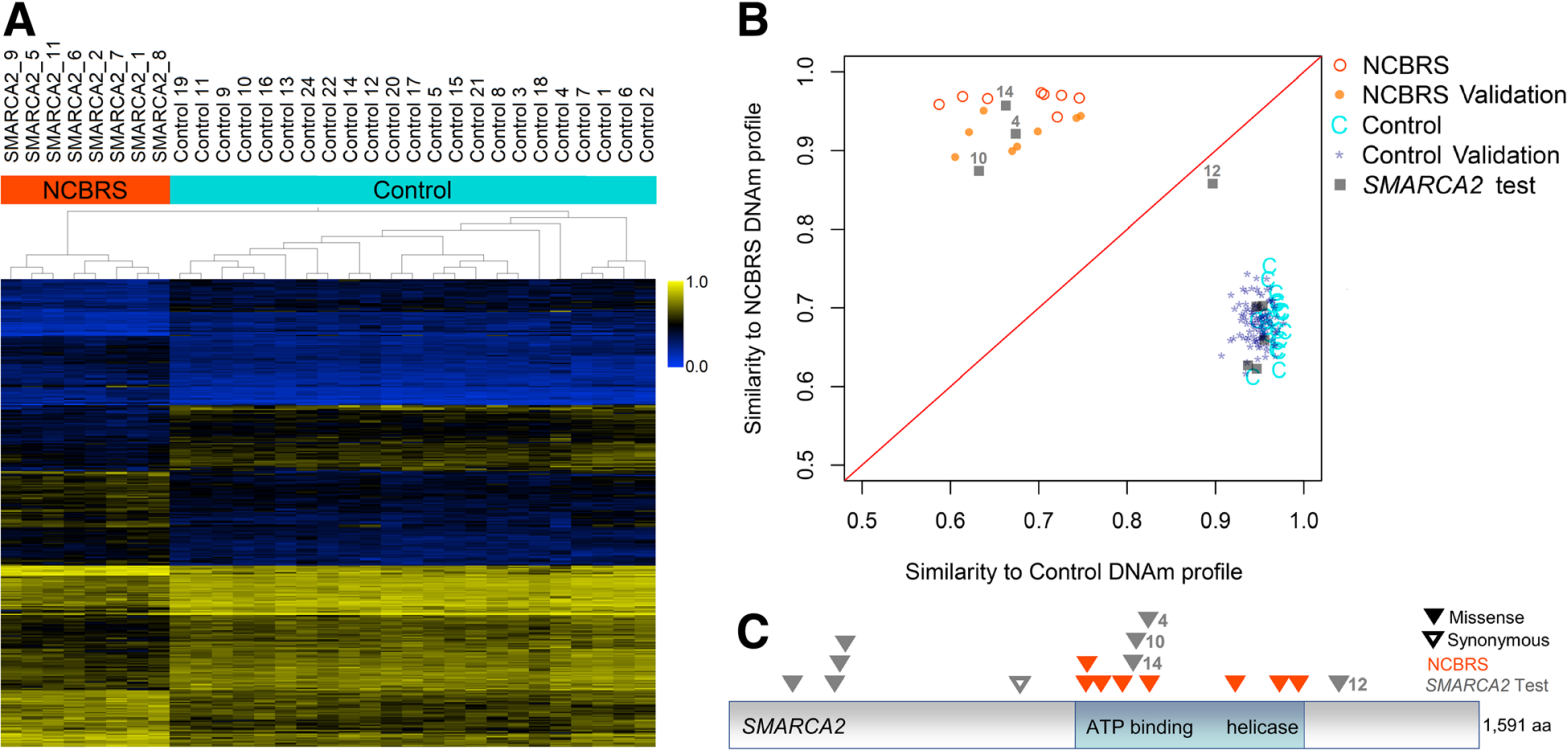
NCBRS-*SMARCA2* DNAm signature classifies variants of uncertain significance**. a** The heatmap shows the hierarchical clustering of NCBRS cases (*n* = 8) and age- and sex-matched neurotypical controls (*n* = 23) using 429 differentially methylated CpG sites specific to *SMARCA2* pathogenic variants. The color gradient indicates the β (DNAm) value ranging from 0.0 (blue) to 1.0 (yellow). DNAm profiles fall into two separate clusters corresponding to NCBRS cases (orange) and controls (cyan). Euclidean distance metric is used in the clustering dendrogram. **b** Classification model based on DNAm signature. The median-methylation profile for signature-derivation NCBRS cases (n = 8) and controls (n = 23) were calculated at the CpG sites comprising the NCBRS-*SMARCA2* DNAm signature. The Pearson correlation of each sample with the median profile of controls and that of NCBRS cases are plotted on the x- and y-axes respectively. The difference of these correlations constitute the NCBRS-*SMARCA2* score. Positive NCBRS-*SMARCA2* scores (pathogenic) fall above the decision boundary (red line) and negative (benign) fall below it. Additional neurotypical control whole-blood samples (*n* = 94; Control Validation) all classified as benign. Additional NCBRS cases with pathogenic *SMARCA2* variants (n = 8; NCBRS Validation; GSE116992) classified as pathogenic. *SMARCA2* variant test cases (n = 9; grey squares with SMARCA2_IDs denoted) were scored: three were classified as pathogenic (SMARCA2_4, SMARCA2_10, and SMARCA2_14), five were classified as benign (SMARCA2_15–19, IDs not shown), and one was classified as benign but its score was near 0, falling close to the decision boundary (SMARCA2_12). **c** Schematic of the *SMARCA2* amino acid sequence with NCBRS signature cases and *SMARCA2* test variants indicated. Numeric labels indicate sample IDs corresponding to those in (**b**)

To define a gene-specific DNAm profile of *SMARCA2* variants associated with NCBRS, we compared genome-wide DNAm in NCBRS patients harboring pathogenic *SMARCA2* sequence variants, according to ACMG guidelines (n = 8), with matched neurotypical controls (*n* = 23; Fig. 1). A DNAm signature of 429 significantly differentially methylated CpG sites was identified (adjusted *p*-value< 0.05, |Δβ| < 10% [10% methylation difference]; Additional file 2: Table S4). Hierarchical clustering of DNAm values at the signature sites clearly distinguished NCBRS cases from neurotypical controls (Fig. 2).

### *SMARCA2* variant classification

We derived an NCBRS-*SMARCA2* score based on the DNAm signature to classify a validation cohort of independent cases and controls as well as *SMARCA2* query variants (Additional file 2: Table S6). Negative scores were assigned to 94/94 independent neurotypical pediatric controls classifying them as benign, demonstrating 100% specificity of the signature (Fig. 2). Positive scores were assigned to 8/8 independent NCBRS cases with different pathogenic variants in the ATPase/helicase domain from a previous study [34], demonstrating 100% sensitivity of the signature. Three VUS in *SMARCA2* were assigned positive scores, classifying them as “pathogenic” using our DNAm model (Fig. 2). Five *SMARCA2* variant samples demonstrated negative scores classifying them as “benign” using our DNAm model (Fig. 2).

One sample from a patient with a clinical diagnosis of NCBRS (but only mild neurodevelopmental issues) and a VUS distal to the ATPase/helicase domain (SMARCA2_12) was assigned a model score less than 0 (classifying as “benign”); however, the sample did not clearly cluster with either cases or controls (Fig. 2). This sample demonstrated a DNAm profile between that of NCBRS cases and controls (Fig. 2). The unique DNAm profile of SMARCA2_12 arises from the fact that at some CpG sites DNAm aligns with NCBRS cases whereas at others it aligns with controls (Additional file 1: Figure S1). We defined subsets of CpG sites at which the DNAm value SMARCA2_12 was typical of either controls or NCBRS cases in this study. To include signature CpG sites conservatively, we restricted defining sites similar to NCBRS cases as those within the range of β-values observed in the signature-derivation NCBRS cases and not in the range of controls (*n* = 106; Additional file 2: Table S4). Similarly, control-overlapping sites were defined as those within the observed range of signature-derivation control β-values and not the range of NCBRS cases (*n* = 204; Additional file 2: Table S4). At 204/429 (48%) of the signature CpG sites the β-value of SMARCA2_12 overlapped controls in that they were both within the control range and outside of the NCBRS range (Additional file 2: Table S4). At 118/429 (28%) of the signature sites the β-value of SMARCA2_12 overlapped the NCBRS methylation values. Gene ontology analysis of these CpG sites is presented below. No overlap of DNAm status between either cases or controls occurred at 21/429 (5%) signature sites for SMARCA2_12 (Additional file 2: Table S4). At the remaining 86/429 (20%) sites, DNAm status of SMARCA2_12 overlapped both NCBRS and control β-value ranges. There were no differences in the proportion of hyper- vs. hypo-methylated probes; however, DNAm levels across probes in the same gene tended overlap with either cases or controls (Additional file 2: Table S4; Additional file 1: Figure S2).

### Variant classification is independent of blood cell type composition and age

We assessed our samples for possible differences in blood cell proportions using our DNAm data. We found a significant (*p* < 0.001, Students t-test) reduction in predicted CD4+ T cell proportion in the NCBRS cases used to derive the signature versus signature controls and a significant increase (*p* < 0.05, Students t-test) in predicted monocyte proportion (Additional file 1: Table S3). Therefore, we accounted for monocyte cell proportion in our regression model. To further ensure that signature classification was not affected by cell-type proportion, we used the NCBRS-*SMARCA2* score to classify DNAm data from sorted cell populations; all cell types were assigned negative scores paralleling whole-blood controls more than NCBRS cases (Additional file 1: Figure S2). Since the NCBRS-*SMARCA2* signature was derived using cases and controls greater than 3 years of age, we classified four additional whole-blood samples from one-year-old controls; all were assigned negative scores classifying as benign (Additional file 1: Figure S3). We also scored DNAm data for three technical replicates that were run using the same DNA sample in a different batch of reagents and microarrays: two cases used to derive the signature (SMARCA2_1 SMARCA2_2) and one test variant (SMARCA2_4). All three demonstrated highly similar scores differing by less than 1% from their replicate samples (Additional file 2: Table S6; Additional file 1: Figure S3).

### Genes with differential DNAm in the *SMARCA2*-specific signature

The 429 CpG sites in the NCBRS-*SMARCA2* DNAm signature were located in the promoters or gene bodies of 225 RefSeq genes (Additional file 2: Table S4). We performed gene ontology analysis of the 429 signature sites using GREAT [35]. GREAT identified 547 genes associated with the 429 CpG sites. We assessed GO terms enriched in the signature CpG sites for molecular function (Additional file 2: Table S7), biological processes (Additional file 2: Table S8), cellular components (Additional file 2: Table S9), and human phenotypes (Additional file 2: Table S10). Pathways and processes involved in brain function/development as well as cellular growth and development were identified across these GO analyses. Finally, we performed an additional analysis looking for differentially methylated regions (DMRs) in the signature derivation cases versus controls using bumphunting [36] which defines consistent patterns of DNAm gain or loss in the vicinity of several genes. The top hits included *GJA8, CACNA1H,* and *HCG4P6* (Additional file 2: Table S11).

Next, we assessed the GO terms enriched for by the NCBRS-*SMARCA2* CpG sites where SMARCA2_12 (the patient with the intermediate classification score) was typical of NCBRS cases (*n* = 106) and controls (*n* = 204; Additional file 2: Table S4). The NCBRS-typical probe list was enriched for few GO terms (Additional file 2: Table S12); an enriched term was related to digital abnormalities (“Short middle phalanx of the 5th finger”); this term was also enriched in the NCBRS-*SMARCA2*-signature (Additional file 2: Table S10). SMARCA2_12 demonstrates digital abnormalities typical of NCBRS (Additional file 1: Figure S1). CpGs overlapping Runt-related transcription factor 2 (*RUNX2*) in part implicated these terms (Additional file 1: Figure S2). The only enriched cellular component, “fascia adherens”. The control-overlapping CpGs in SMARCA2_12’s DNAm profile were enriched for many of the same GO terms as the NCBRS-*SMARCA2* signature, including Wnt signaling (ex. B Cell CLL/Lymphoma 9 Like [*BCL9L*]; Additional file 1: Figure S2) and cell adhesion and synaptic components (Additional file 2: Table S13) all relevant to neurodevelopment. We also compared each list of genes to which the control- and NCBRS-overlapping CpGs map with the SFARI ASD gene list. There was a significant (*p* < 0.001, Chi-square test) enrichment of the SFARI ASD genes in the control-overlapping gene list with 12/106 genes shared, while there was non-significant enrichment for ASD genes in the NCBRS-overlapping gene list, with 6/66 genes shared.

### DNAm validation by sodium bisulfite pyrosequencing

**Fig. 3:**
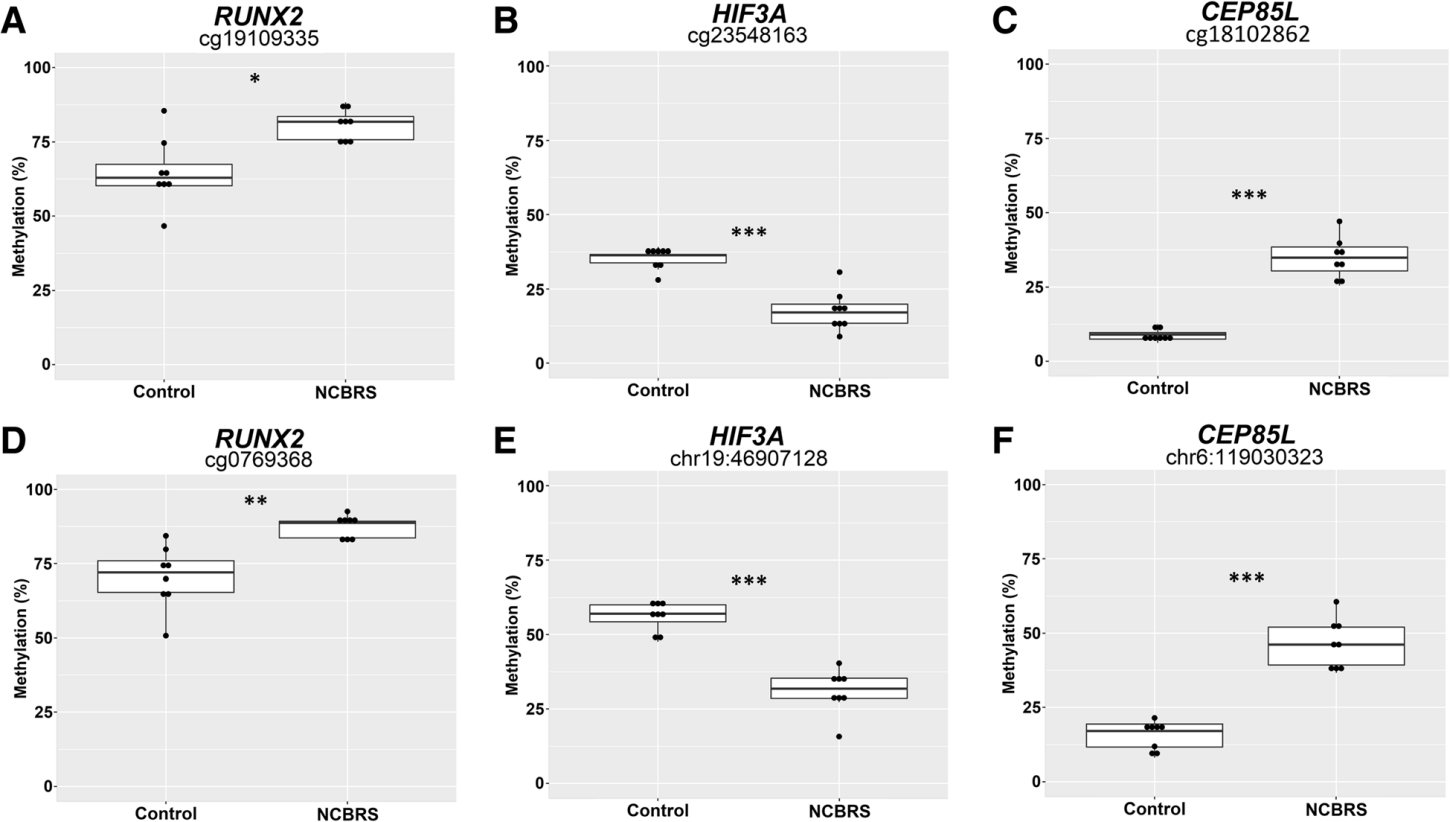
Targeted sodium bisulfite pyrosequencing validation of DNAm alterations in NCBRS-*SMARCA2* signature cases**. a**-**c** DNAm was assessed for three sites in the DNAm signature in the promoters of *RUNX2* (cg19109335), *HIF3A* (cg23548163), and *CEP85L* (cg18102862); the change in DNAm across these sites was: + 17%, + 26%, and − 19% respectively. **d**-**f** Additional neighboring CpG sites covered by the assays from **a**-**c**. The additional CpG site in the *RUNX2* promoter is represented on the EPIC array, those in HIF3A and CEP85L are not; the change in DNAm across these sites was: + 16%, + 30%, and − 25% respectively. Statistical significance between NCBRS and control groups was assessed using a Student’s t-test, *p*-values were corrected for multiple CpG assessed *p < 0.05, **p < 0.01, ***p < 0.001

We selected DNAm changes in the promoters of three genes in the DNAm signature for validation by bisulfite pyrosequencing (Fig. 3). We selected CpGs overlapping *RUNX2,* Centrosomal Protein 85 Like (*CEP85L*)*,* and Hypoxia Inducible Factor 3 (*HIF3A*) based on three criteria: CpG located in the promoter/5’UTR of the gene, the potential relevance of the gene to the NCBRS phenotype, and a |Δβ| > 15%. Each assay also covered one other CpG site which was not in the signature for a total of six CpGs sites assessed. All six CpGs demonstrated a significant DNAm change in the signature cases versus matched controls (Fig. 3). The CpG cg19109335 was identified as differentially methylated between cases and controls while an adjacent CpG also covered by the pyrosequencing assay, cg07069368, was not. The cg19109335 site was validated to have the same direction and similar magnitude of DNAm change as determined by the microarray; cg07069368 was also differentially methylated in the pyrosequencing assay, and had a very similar profile to cg19109335 (Fig. 3); both of these displayed increased DNAm in NCBRS cases. Using another pyrosequencing assay, we also validated reduced DNAm of cg23548163 in the 5’UTR of *HIF3A.* This pyrosequencing assay also covered a CpG at chr19:46807128 which also demonstrated reduced DNAm in NCBRS cases (Fig. 3). Finally, we validated increased DNAm of cg18102862 in *CEP85L.* The assay also included a CpG at chr6:119030323 which demonstrated increased DNAm in NCBRS cases. *CEP85L* encodes Centrosomal Protein 85 Like.

## Discussion

Genome-wide analysis identified a set of changes in DNAm (DNAm signature) associated with pathogenic *SMARCA2* variants in the peripheral blood of patients with NCBRS. The signature allows for classification of *SMARCA2* missense variants in concordance with the clinical phenotype and predicted pathogenicity of the variant.

Three *SMARCA2* VUS samples were assigned positive model scores classifying them as “pathogenic”. Each of these samples (IDs: SMARCA2_4, SMARCA2_10, and SMARCA2_14) has a missense variant in the *SMARCA2* ATPase domain predicted to be damaging based on in silico tools (Table 1) [17, 38]. Each of these patients also has a clinical diagnosis of NCBRS and typical phenotypic features consistent with the disorder including sparse hair, typical facial dysmorphism, and intellectual disability (ID; Additional file 1: Table S14). Thus, the genome-wide DNAm profiling using the signature allows for molecular confirmation in individuals with ambiguous/uncertain diagnostic testing results. Five patient samples were assigned negative models scores classifying them as “benign” (Fig. 2). None of these individuals have features of NCBRS and their variants are proximal to the ATPase domain. Three of these variants (SMARCA2_16, SMARCA2_17, SMARCA2_18) were predicted to be benign based in silico tools (Table 1) one was a synonymous variant (SMARCA2_19) and one was a VUS (SMARCA2_15; Fig. 2) predicted to be “potentially damaging” by in silico tools (Table 1). The classification of this VUS as benign using the NCBRS-*SMARCA2* DNAm signature highlights the difficulty in relying purely on in silico prediction tools.

One of the *SMARCA2* test variant samples (SMARCA2_12) was obtained from a patient with a VUS distal to the ATPase/helicase domain. This patient is a 16-year-old female with learning disability and a subset of facial and developmental characteristics consistent with NCBRS (Additional file 2: Table S1); her mild neurodevelopmental features are atypical. The *SMARCA2* VUS she carries occurs 77 amino acids distal to the C-terminal end of the ATPase/helicase domain located in exon 27 (Table 1; Fig. 2). To our knowledge, this is the first report of a patient with a clinical NCBRS diagnosis and a variant in this exon. There have been three other reports of patients with neurodevelopmental abnormalities and variants distal to the *SMARCA2* ATPase/helicase. Two of these variants were proximal to that of SMARCA_12 (i.e. closer to the ATPase/helicase domain); one just outside the domain [24], the other approximately 30 amino acids distal to it [23]. Both of these patients are described to have a typical NCBRS phenotype (Additional file 1: Table S14). The third patient had a variant in the *SMARCA2* bromo domain. This patient was described to have a “distinct but overlapping phenotype with NCBRS”; overlapping features included ID, seizures, absent speech, and slight prominence of interphalangeal joints (Additional file 1: Table S14) [39]. The phenotype of these four patients demonstrate variable overlap with the typical NCBRS features suggesting that the boundary of the ATPase/helicase domain does not strictly define NCBRS etiology. Therefore the DNAm profile of individuals with features of NCBRS and genomic variants distal to this domain will be particularly interesting to study. We expect that functional classification tools including DNAm signatures will be ideally suited to aid in understanding the phenotypic impact of these variants. These DNAm data challenge clinical diagnosis of SMARCA2_12 as NCBRS. Further, they call into question the issue of definitive criteria for clinical diagnosis of NCBRS. They also raise the question of whether variants outside the ATPase/helicase domain cause NCBRS or an NCBRS-like phenotype. The patient with the bromo domain variant is reasonably defined as related to but not NCBRS based on phenotypic data; however, in light of her atypical phenotype and partial DNAm signature profile, the appropriateness of the clinical classification of NCBRS for SMARCA2_12 is not as obvious.

GO analysis on the CpG sites comprise the NCBRS-*SMARCA2* signature identified genes related to NCBRS pathophysiology. Many genes and processes involved in brain function/development were identified, relevant to the high frequency and degree of ID observed in NCBRS. There was enrichment of several calcium channel and synaptic function GO terms in the DNAm signature (Additional file 2: Table S7 and Additional file 2: Table S8). Further, there was significant (*p* < 0.001, Chi-square test) enrichment of ASD-associated genes with 10% (22/225) of signature-overlapping genes present in SFARI (Simons Foundation Autism Research Initiative). Individuals with NCBRS often display ASD-like features as noted in our cohort and others [38]. We also looked for genes overlapping the DNAm signature with known roles in ID. Using a curated list of 484 genes directly implicated in ID (ID Project, University of Colorado Denver) we found two genes: KN Motif And Ankyrin Repeat Domains 1 (*KANK1*)*,* associated with cerebral palsy [40], and Transcription factor 4 (*TCF4*)*,* associated with Pitt-Hopkins Syndrome [41]. Alterations in DNAm at these ASD and ID genes suggest differential regulation in these individuals, contributing to neurodevelopmental aberrations in NCBRS. One signature CpG also overlapped Calcium Voltage-Gated Channel Subunit Alpha1 H (*CACNA1H*), which also had a DMR identified using bumphunting. Variants in *CACNA1H* are associated with seizure risk, a key component of NCBRS [42]. Missense variants in this gene are also implicated in ASD [43]. The enrichment of neurodevelopmental GO terms recapitulates a key finding from a previous DNAm study of NCBRS [34]. Together, these results demonstrate that neurodevelopmental genes are disproportionately represented in DNAm changes associated with NCBRS.

The NCBRS-*SMARCA2* DNAm signature also overlaps genes related to growth and development of various cell/tissues types. Several GO biological processes involved in hematopoietic cell differentiation were enriched. These were due in part to reduced methylation at Homeobox B4 (*HOXB4*). *HOX* genes encode key regulators of early transcriptional programs governing stem cell differentiation [44]. Wnt pathway genes were also enriched (Additional file 2: Table S8); these are downstream targets of HOX regulation and important for differentiation of hematopoietic stem cells into blood cells [45]. Persistent aberrant DNAm of *HOXB4* and Wnt genes in NCBRS cases may indicate alterations in the regulation of stem cell differentiation during development. The longest DMR identified overlapped Gap Junction Protein Alpha 8 (*GJA8*) which encodes a transmembrane connexin protein involved in lens growth and maturation of lens fiber cells [46]. Growth and development GO terms were also highly enriched in a previously reported NCBRS DNAm study [34], though this study did not identify Wnt pathway signaling genes.

The NCBRS-*SMARCA2* signature shares relatively few CpGs with other epigene-specific DNAm signatures. We screened for overlapping CpG sites (same site and direction of methylation change) between published epigene signatures developed on the Illumina 450 k array and the 213 NCBRS-*SMARCA2* signature sites on the 450 k array: 17/7085 (0.2%) overlap with the *NSD1* signature [6], 2/113 (1.8%) with the *KMT2D* signature [8], and 2/103 (1.9%) the *CHD8* signature [10]. There were no overlaps with the *CHD7* or 16p11.2 deletion signatures. Not surprisingly, we found more overlap between the NCBRS-*SMARCA2* signature and signatures for other genes in the BAF complex (also generated on the Illumina EPIC array) with 8/135 (5.9%) shared with the *SMARCB1* signature and 6/146 (4%) with the *ARID1B* signature [34]. This is consistent with findings from a recent study showing that BAF complex genes have overlapping DNAm profiles [34]. While these overlapping CpGs may be biologically relevant, they represent a small percentage of the number of signature probes generally required for robust variant classification.

We validated six total CpG sites from the promoter regions of three genes in the using sodium bisulfite pyrosequencing. Two CpG sites were validated to have increased DNAm in NCBRS cases in the *RUNX2* promoter. *RUNX2* encodes a transcription factor involved in osteoblast differentiation and cartilage hypertrophy [47]. Pathogenic variants in *RUNX2* have been implicated in skeletal disorders such as cleidocranial dysplasia, dental anomalies, and brachydactyly [48, 49]. In human phenotype GO analysis, changes in *RUNX2* enriched for abnormalities of the fifth finger and dental abnormalities (Additional file 2: Table S10). Differential methylation of *RUNX2* is interesting in the context of NCBRS given the clinical features of prominent interphalangeal joints, delayed dental eruption, and oligodontia in this condition. SMARCA2_12 retained an NCBRS-overlapping methylation pattern at this gene, consistent with her digital and craniofacial abnormalities typical of the disorder.

Next, we validated decreased DNAm at two CpG sites in the *HIF3A* promoter. *HIF3A* encodes the transcription factor Hypoxia Inducible Factor 3 Subunit Alpha. Changes in regulation of hypoxia-inducible gene expression during fetal development are associated with altered neurodevelopment, and implicated in several neurodevelopmental disorders [49, 50]. DNAm of *HIF3A* appears to be functionally important for growth, as it is associated with body-mass index [51]. Finally, we validated increased DNAm in the promoter of *CEP85L*. Beyond the fact that CEP85L acts as a breast cancer antigen, little is known about the function of this protein. Notably, the DNAm level of both *CEP85L* CpG sites did not overlap between signature cases and controls (Fig. 3).

In line with her atypical NCBRS clinical phenotype, we found that SMARCA2_12 had a partial NCBRS-*SMARCA2* DNAm signature with a classification score intermediate between NCBRS cases and controls. At some of the signature sites, her methylation level was characteristic of NCBRS DNAm values, while at others it was characteristic of controls. The DNAm profile of this patient may reflect partial functional impairment of the SMARCA2 protein, leading to partial NCBRS molecular/cellular changes during development and a milder phenotype. We performed GO analysis on the CpGs comprising these two subsets of sites. Using the NCBRS-overlapping sites, we found that enrichment of terms related to digital abnormalities. SMARCA2_12 displays prominent interphalangeal joints, short metacarpals, and facial dysmorphology characteristic of NCBRS. The only enriched cellular component, “fascia adherens” contained the gene Junction Plakoglobin (*JUP*) for which variants are associated with disorders with hair abnormalities [52]. Interestingly, SMARCA2_12 is noted to have coarse hair quality. The genes overlapping the DNAm levels of controls were very similar to the complete NCBRS-*SMARCA2* signature, containing many neurological, cell adhesion, and synaptic GO terms (Additional file 2: Table S13). Thus, SMARCA2_12 does not have the DNAm alterations observed at neurological genes in other NCBRS patients; this is consistent with her clinical phenotype. Further, for SMARCA2_12, the CpGs at the genomic sites enriched for SFARI ASD genes demonstrated levels of DNAm parallel to controls and not NCBRS cases. This indicates that at genes relevant to ASD, SMARCA2_12 does not have the DNAm changes typical of other NCBRS cases. In summary, the DNAm profile of SMARCA2_12 is disproportionally similar to controls at genes involved in neurodevelopment in contrast to the altered methylation signals at these sites in all other NCBRS cases. These results are consistent with the milder neurocognitive deficit of this patient compared to most NCBRS patients. The concordance of the DNAm profile of SMARCA2_12 with her clinical phenotype supports the view that DNAm changes in NCBRS are functionally relevant to pathophysiology of this disorder.

We propose that the DNAm signature is a better tool for NCBRS diagnosis than relying on the location of the variant in the gene. We demonstrate that a patient with a variant most distal to the ATPase domain with an atypical NCBRS clinical presentation has a partial NCBRS-*SMARCA2* DNAm signature profile. It may be that the degree of clinical overlap of the three other cases with variants distal to the ATPase/helicase domain will also be reflected in their concordance with the DNAm signature. That is, cases with more typical NCBRS features and variants near the ATPase/helicase domain may classify as pathogenic. The variant in the *SMARCA2* bromo domain may be associated with its own unique DNAm signature, and thus its degree of overlap with the NCBRS-*SMARCA2* signature is difficult to predict. Scoring of such rare cases using our model would greatly aid in establishing genotype-epigenotype-phenotype correlations for NCBRS. We propose that the NCBRS-*SMARCA2* DNAm signature can be used to aid clinical diagnosis and quantify the overlap of patients with ambiguous phenotypes with typical NCBRS. Because of the added complexity of partial DNAm profiles, implementation of our DNAm signature score as a clinical test would require moving from a binary classification system to a scoring system. At this time, one intermediate sample is not sufficient to develop such a system. Again, scoring additional patients with variants distal to the *SMARCA2* ATPase/helicase domain using our approach will be necessary.

The DNAm data we present suggest a genotype-epigenotype-phenotype correlation for *SMARCA2* variants in NCBRS. Pathogenic variants within the ATPase/helicase domain lead to a specific DNAm signature associated with classic NCBRS clinical features. Variants proximal to the domain are not associated with the DNAm signature or NCBRS clinical features. A variant just distal to the ATPase/helicase domain is associated with a partial DNAm signature and a mild/atypical NCBRS clinical phenotype. These finding are important for understanding NCBRS pathophysiology, but are also applicable to generating other gene-specific DNAm signatures moving forward. These data are the first report of a DNAm signature that is associated with a specific protein domain. This is most likely due to the specificity of variants in NCBRS for the ATPase/helicase domain; previous signatures were derived for conditions associated with mostly loss-of-function variants/deletions occurring across the gene [1, 6–9, 11]. In light of the domain specificity of the NCBRS-*SMARCA2* signature, moving forward careful consideration should be paid to the selection of samples for generation of gene-specific signatures, especially when pathogenic variants are known to be concentrated in specific domains. Further, the findings we present here demonstrate the importance of detailed clinical data in both choosing samples to generate DNAm signatures and in interpreting DNAm signature classifications.

## Conclusions

In conclusion, we report a DNAm signature for NCBRS-associated *SMARCA2* pathogenic missense variants that can be used to classify VUS in *SMARCA2*. The DNAm changes in the NCBRS-*SMARCA2* DNAm signature occur in genes that represent novel and highly specific targets for future studies to elucidate the molecular pathophysiology of NCBRS and inform the development of targeted therapies, especially with respect to neurodevelopment. We report an NCBRS case with a *SMARCA2* variant distal to the ATPase/helicase domain with a mild clinical (especially neurodevelopmental) features who demonstrates a partial NCBRS-*SMARCA2* DNAm signature. The DNAm profile at genes where this patient resembles other NCBRS patients is consistent with her clinical phenotype. These findings provide novel insight into the functional relevance of DNAm signatures, specifically with regard to the location of variants within the gene and concordance with clinical phenotype. Use of this DNAm signature for assessing more patients with variants outside the *SMARCA2* ATPase/helicase domain will allow refinement of the classification model and better definition of genotype-phenotype correlations in NCBRS. Taken together, these data provide the foundation for DNAm-based diagnostics, novel insights into NCBRS pathophysiology, and a platform for developing new therapies.

## Additional files

Additional file 1: Supplementary Figures and Tables (**Figure S1–S3** and **Tables S5** and **S14**). (DOCX 1012 kb)

Additional file 2: Supplementary Figures and Tables (**Tables S1–S4,** and **S6–S13**). (XLSX 183 kb)

## Data Availability

The microarray datasets generated and analyzed during the current study are available in the GEO repository, GSE125367

## Abbreviations

ADHD: Attention deficit hyperactivity disorder
ASD: Autism spectrum disorder
BAF: BRG1- or HBRM-associated factors
bp: base pair
CADD: Combined annotation dependent depletion
CSS: Coffin-Siris syndrome
DMR: Differentially methylated region
DNAm: DNA methylation
ExAC: Exome Aggregation Consortium
FACS: Fluorescence activated cell sorting
GEO: Gene expression omnibus
GO: Gene ontology
ID: Intellectual disability
NCBRS: Nicolaides-Baraitser syndrome
REB: Research ethics board
SFARI: Simons Foundation Autism Research Initiative
UTR: Untranslated region
VUS: Variant of uncertain significance
