# Supplementary material for: New insights into DNA methylation signatures: SMARCA2 variants in Nicolaides-Baraitser syndrome
Source: BMC Med Genomics. 2019 Jul 9;12:105. doi: 10.1186/s12920-019-0555-y (PMC6617651; doi:10.1186/s12920-019-0555-y)
Supplement: Supplementary file 1 — Supplementary Figures and Tables (Figure S1–S3 and Tables S5 and S14). (DOCX 1012 kb) [file 12920_2019_555_MOESM1_ESM.docx]

**Supplemental Figures**


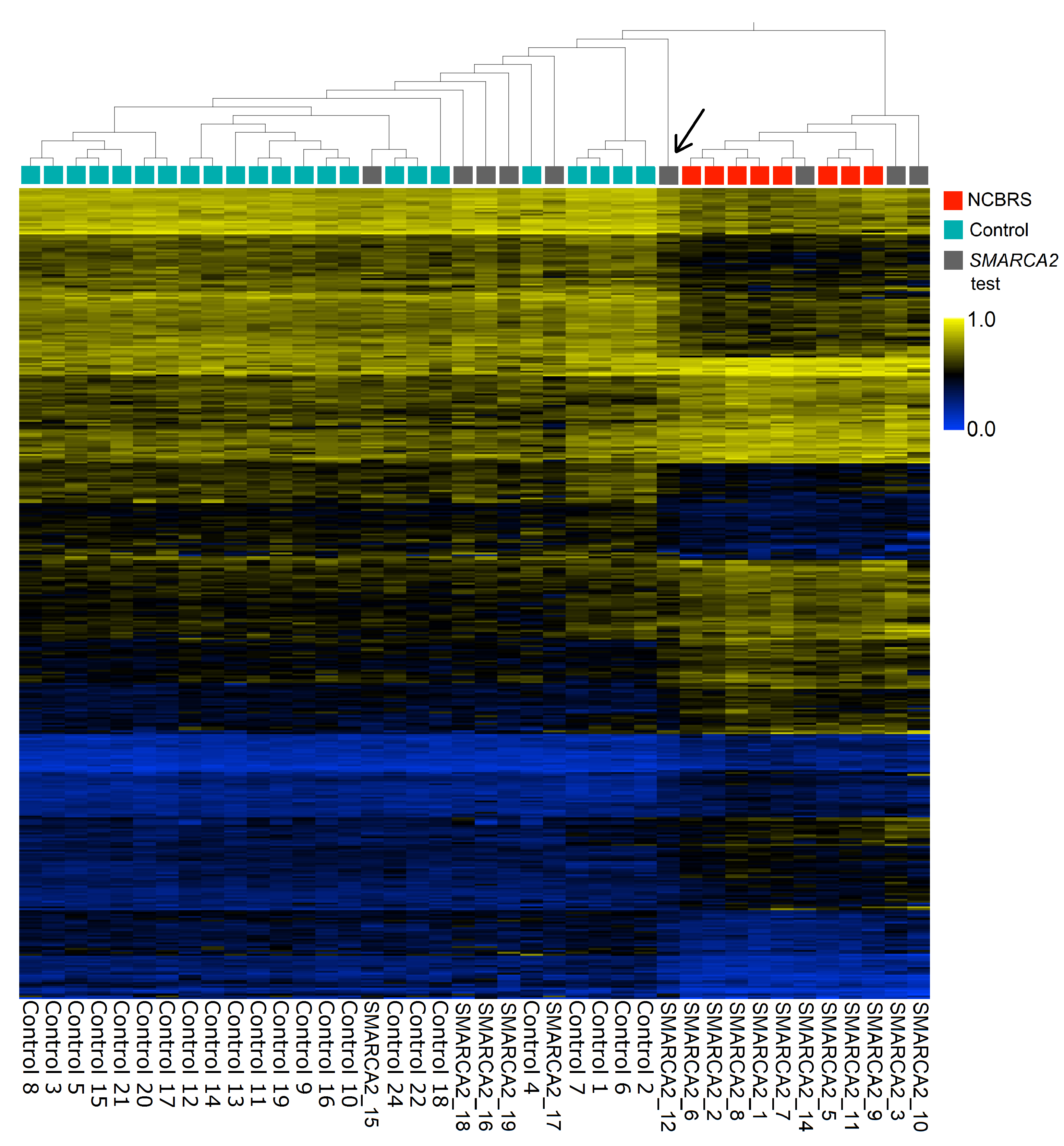


**Figure S1. Hierarchical clustering of *SMARCA2* variants at the NCBRS-SMARCA2 DNAm signature sites**. The color gradient indicates the raw β (DNAm) level of each probe from 0.0 (blue) to 1.0 (yellow). DNAm profiles fall into two separate clusters corresponding to signature-derivation NCBRS cases (n=8; orange) and controls (n=23; cyan). Most test variants (grey) cluster with either cases or controls, except sample SMARCA2_12 (black arrow) which demonstrates an intermediate DNAm profile. Euclidian distance metric is used for the clustering dendogram.


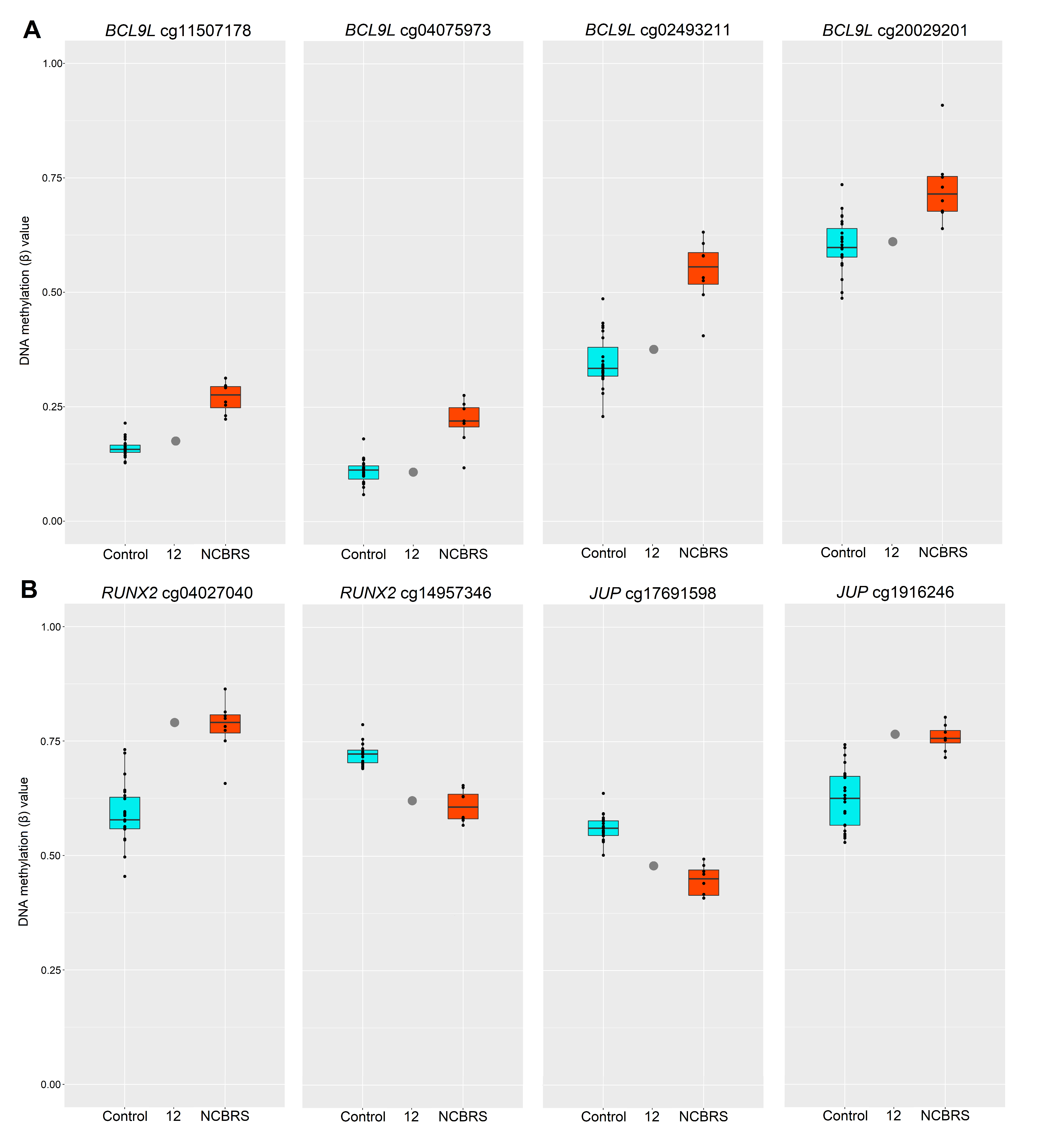


**Figure S2. Control- and NCBRS-overlapping DNA methylation profile of SMARCA2_12 at multiple DNAm signature CpGs overlapping the same genes**. Box plots of DNAm (β) values for controls (cyan) and NCBRS cases (orange) used to generate the DNAm signature. The β-value for the intermediate SMARCA2_12 case is shown (dark grey circled). (A) Top panel show four contiguous CpG sites from the Wnt pathway gene *BCL9L* at which SMARCA2_12 resembles controls more than cases. (B) The bottom panel shows CpG sites at which SMARCA2_12 resembles NCBRS cases more than controls: *RUNX2* from the “Short middle phalanx of the 5th finger” GO term and *JUP* from the “fascia adherens” GO term. Data are box plots with individual data points shown.


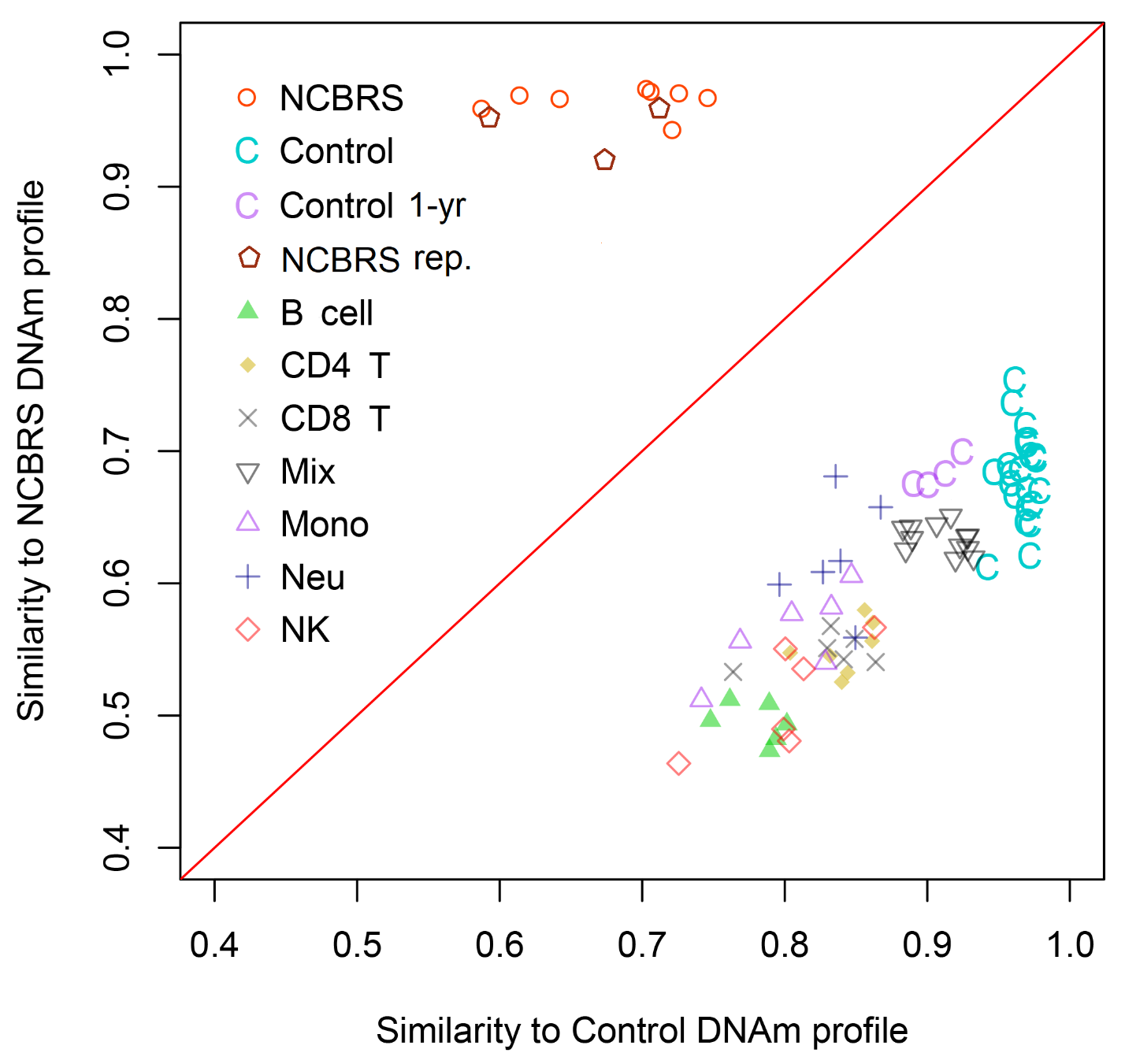


**Figure S3. Classification of purified blood cell types and young controls using the NCBRS-SMARCA2 DNAm signature.** DNA methylation EPIC data from six sorted blood cell types (CD19+ B cells, CD4+ T cells, CD8+ T cells, CD14+ monocytes [Mono], neutrophils [Neu] CD56+ NK cells) as well as a mixture of the six (mix) classified using the NCBRS-SMARCA2 score (n=6 for each). All were assigned negative scores classifying as benign. Four one-year-old neurotypical control whole-blood samples (Control 1-yr) were also scored, demonstrating negative NCBRS-SMARCA2 scores and classifying as benign. Technical replicates of two NCBRS signature cases and one NCBRS test sample (NCBRS rep.) scored very similarly to their replicate samples.

**Supplemental Tables**

**Table S5.** Primers used for pyrosequencing assays**.**

| ***RUNX2*** |  | PCR product size (bp) |
| --- | --- | --- |
| RUNX2-F1 | TGTGTGTAGTAAGGAATAATGTTTATAGAT | 97 |
| RUNX2-R1 | CGCCAGGGTTTTCCCAGTCACGACTTTCCCAAAAATATTTCCA ATTTTCTTCT |  |
| RUNX2-S1 | ATGTTTATAGATAGAGTATGTTAG |  |
| ***CEP85L*** |  | 272 |
| CEP85L-F1 | GGGATTGATAGTTTATAGAGAAATAAGAG |  |
| CEP85L-R1 | CGCCAGGGTTTTCCCAGTCACGACACAAAAACTAACAAAAATA TTTCCTCTAA |  |
| CEP85L-S1 | TTTAATTGTTGTTATTTAAGGAAAA |  |
| ***HIF3A*** |  | 174 |
| HIF3A-F1 | CGCCAGGGTTTTCCCAGTCACGACGGGTTTTATAAGTGATAGATT |  |
| HIF3A-R1 | CCCCTCCTCCCAAAAACCTTATTCT |  |
| HIF3A-S1 | ACCCCCCCCCCTAAT |  |

**Table S14.** Genotype and phenotype data for reported cases with variants in *SMARCA2* distal to the ATPase/helicase domain.

| Case | Variant | Position | Diagnosis | Phenotype summary |
| --- | --- | --- | --- | --- |
| Bramswig 2015, *Hum Genet* (1) | c.3655G>C, p.Ala1219Pro | Exon 25 | NCBRS | Typical facial NCBRS features (triangular shape of face with malar flattening, downslanted palpebral fissures, hypertelorism, a broad nasal root, long and flat philtrum), sparse hair, “hyperactive”, no seizures, brachytelephalangy and short fingernails. Broad feet with no hypoplasia of the toenails |
| Tang 2017 *Am J Med Gent* (2) | c.3721C>G, p.Gln1241Glu | Exon 26 | NCBRS | NCBRS-specific craniofacial features, reduced speech, intellectual disability, autism, and seizures |
| SMARCA2_12 | c.3849G>T, p.Trp1283Cys | Exon 27 | NCBRS | Normal growth parameters, learning disability and attention deficit-hyperactivity disorder, enrolled to begin college with good social functioning, facial coarsening with full lips, a wide mouth and lower lip eversion. Hair was not sparse but rather slow-growing, curly and coarse in quality |
| Sousa 2014 *Am J Med Genet  C* (3) | c.4258G>A, p.Gly1420Arg | Bromo domain | ID | Non-NCBRS features (no sparse hair or typical face, no independent walking and severe feeding problems) but still demonstrates overlap with NCBRS (severe ID, seizures, absent speech, rounded pre‐maxilla, decreased subcutaneous fat, and slight prominence of interphalangeal joints in the second decade of life) |

*SMARCA2* helicase domain ends at p.1211

Supplemental References

1. Bramswig NC, Ludecke HJ, Alanay Y, Albrecht B, Barthelmie A, Boduroglu K, et al. Exome sequencing unravels unexpected differential diagnoses in individuals with the tentative diagnosis of Coffin-Siris and Nicolaides-Baraitser syndromes. Human genetics. 2015;134(6):553-68.

2. Tang S, Hughes E, Lascelles K, Simpson MA, Pal DK. New SMARCA2 mutation in a patient with Nicolaides-Baraitser syndrome and myoclonic astatic epilepsy. American journal of medical genetics Part A. 2017;173(1):195-9.

3. Sousa SB, Hennekam RC. Phenotype and genotype in Nicolaides-Baraitser syndrome. American journal of medical genetics Part C, Seminars in medical genetics. 2014;166c(3):302-14.
